# The Role of Interleukin 17 in Tumour Proliferation, Angiogenesis, and Metastasis

**DOI:** 10.1155/2014/623759

**Published:** 2014-07-07

**Authors:** Bob Yang, Heechan Kang, Anthony Fung, Hailin Zhao, Tianlong Wang, Daqing Ma

**Affiliations:** ^1^Department of Anesthesiology, Xuanwu Hospital, Capital Medical University, Beijing 100053, China; ^2^Anaesthetics, Pain Medicine and Intensive Care, Department of Surgery and Cancer, Faculty of Medicine, Imperial College London, Chelsea & Westminster Hospital, London SW10 9NH, UK

## Abstract

With 7.6 million deaths globally, cancer according to the World Health Organisation is still one of the leading causes of death worldwide. Interleukin 17 (IL-17) is a cytokine produced by Th17 cells, a T helper cell subset developed from an activated CD4+ T-cell. Whilst the importance of IL-17 in human autoimmune disease, inflammation, and pathogen defence reactions has already been established, its potential role in cancer progression still needs to be updated. Interestingly studies have demonstrated that IL-17 plays an intricate role in the pathophysiology of cancer, from tumorigenesis, proliferation, angiogenesis, and metastasis, to adapting the tumour in its ability to confer upon itself both immune, and chemotherapy resistance. This review will look into IL-17 and summarise the current information and data on its role in the pathophysiology of cancer as well as its potential application in the overall management of the disease.

## 1. Introduction

Cancer, according to the World Health Organisation, is still one of the leading causes of death worldwide, accounting for 7.6 million deaths globally. In fact, recent statistics from the United States of America show that one in four deaths in the USA is now caused by cancer [1]. With such numbers, it is prudent therefore to continue finding new and innovative ways in treating this disease. This review will look into interleukin 17 (IL-17) and summarise the current information and data on its role in the pathophysiology of cancer as well as its potential application in the overall management of the disease.

## 2. IL-17: Biology and Function

IL-17 is a cytokine produced by Th17 cells, a T helper cell subset developed from an activated CD4+ T cell [2]. Currently, six IL-17 family members have been identified, IL-17 A through to F. The prototypic family member has been identified as IL-17A, whilst IL-17F shows the highest degree of homology to IL-17A out of all the remaining IL-17 family members [3].

Studies have shown that the key factor stimulating the generation of Th17 cells from naïve T-cells in humans is transforming growth factor-*β* (TGF-*β*), which along with certain inflammatory cytokines (either TGF-*β* with IL-21 or TGF-*β* with IL-6 and IL-23) induces the transcription factor (ROR-gammat) [4, 5]. The activation of ROR-gammat leads then to the development of naïve T-cells to cells which produce IL-17. This process has already been reviewed by Miossec et al. [6].

The main role of IL-17 in humans is in host pathogen defence, in particular to extracellular bacterial and fungi infections. In bacterial infections, the release of IL-17 stimulates a massive inflammatory response, leading to neutrophil accumulation and, in certain intra-abdominal infections, may even end with the formation of abscesses [7]. When IL-17 is missing, susceptibility to extracellular bacterial and fungi infections has been shown to exist.

Studies in IL-17 deficient mice showed increased susceptibility when infected with* Klebsiella pneumonia*,* Toxoplasmosis gondii,* and* Candida albicans* [8–10]. When infected with* Aspergillus fumigatus* and* Candida albicans*, there was more extensive growth of these fungi [11]. In patients with hyper-IgE syndrome, a defect in signal transducer and activator of transcription-3 (STAT3) causes a failure to generate sufficient IL-17 releasing T-cells. The consequent lack of IL-17 has been linked with the high susceptibility of these patients to* Staphylococcus aureus* and* Candida albicans* infections [12, 13]. Whilst the release of IL-17 plays a key role in preventing infection and maintaining health, the deregulation of IL-17 promotes disease, in particular inflammatory conditions such as rheumatoid arthritis and inflammatory bowel disease (IBD). In rheumatoid arthritis, it has been shown that through the activation of T-cells via the IL-17/IL-23 axis, the subsequent Th17 cells become osteoclastogenic, inducing the expression of receptor activator of nuclear factor-*κ*B ligand (RANKL) on both T-cells and osteoblasts, which in turn activates bone destruction and resorption via osteoclasts [14], with IL-17 levels even predictive of damage progression in patients [15].

Interestingly new research suggests another potential pathway for the deregulation of IL-17 expression in arthritis. Whilst previous data has shown that forkhead box protein P3 (Foxp3) expressing T_reg_ cells is key in suppressing immune responses and prevents the development of autoimmune diseases, Komatsu et al. have recently shown that Foxp3^+^ T-cells can actually convert into pathogenic Th17 cells (named, in the study, as exFoxp3 TH17 cells) with arthritogenic and autoreactive properties. This, they conclude, is via the plasticity of Foxp3^+^ T-cells, with unstable Foxp3^+^ T-cells converting to the IL-17 expressing exFoxp3 TH17 cells, thus providing an almost paradoxical pathway on the association between IL-17 and disease [16, 17].

In patients with IBD, it has been consistently shown that their mucosa displays significantly higher levels of IL-17A than normal or ischaemic colitis mucosa, both* in vitro* and* in vivo* [18–21]. In addition, the severity of inflammation and clinical activity corresponded to the degree of IL-17A expression in IBD with no significant difference observed between ulcerative colitis and Crohn's disease, the two main subcategories of IBD [22]. The role of IL-17 is further supported by knockout murine studies of the IL-17 receptor, which showed a subsequent attenuation of the inflammatory effects of IBD [23]. Whilst the importance of IL-17 in human autoimmune disease, inflammation, and pathogen defence reactions has already been established, its potential role in cancer needs to be updated.

### 2.1. IL-17 in Cancer

#### 2.1.1. Tumorigenesis

The role of IL-17 in cancer starts from the initial stages of tumourigenesis having already been established as having a role in the earliest formation of a tumour by its increased presence within the tumour microenvironment [24]. In terms of the protumour role, there is strong evidence stemming from IL-17's role in chronic inflammation as highlighted above. Its protumour role was highlighted in its positive association with increased malignancy of tumours [25]. This is due to the vast accumulation of IL-17 secreting Foxp3^+^ cells in the tumour microenvironment with a dual function of proinflammation and regulation of local T-cell function [26]. This is achieved by blocking the entry of cytotoxic CD8 T-cells and the entry of myeloid derived suppressor cells (MDSCs), thus modifying the local environment and subsequently reducing the local immune response towards the local tumour cells [27]. These MDSCs which enter the environment then respond to the secretion of heat shock protein (HSP) 72 on exosomes from the tumour cells and stimulate the MDSC immune dampening effects [26]. This increased entry of MDSCs and their subsequent function have been associated with a reduced survival in renal cell carcinoma [28]. Despite suppressing antitumour inflammation, IL-17 has also been associated with maintaining the chronic inflammation found either in the preceding condition [29] or within the tumour tissue [30]. This takes place in response to the release of lactate from the tumour cells resulting in increased release of IL-17, in particular IL-17A, via IL-23 dependent and independent pathways [31, 32]. The subsequent rise in IL-17 can go on to trigger the release of proinflammatory cytokine IL-6 and the consequent activation of the STAT3 pathway [33]. This activation of STAT3 has subsequently been linked with the activation of the NF-*κ*B pathway [34] which is associated with the production of proinflammatory cytokines [35]. IL-17, however, has been also shown to activate NF-*κ*B in combination with TNF-*α* via the IL-17B receptor [36, 37]. Alternatively, the tumour cells are also able to perpetuate the local inflammatory response through the expression of CC chemokine ligand 2 (CCL 2) which stimulates entry of IL-17 expressing monocytes which can contribute to the local inflammatory reaction [38]. This is believed to be responsible for the tumour's ability to maintain the proinflammatory reaction locally and also continue to stimulate tumour growth, particularly in prostate cancer [39]. A possible underlying mechanism for this is again through the IL-17 activation of the STAT3 and NF-*κ*B pathways leading to expression of antiapoptotic genes such as Bcl-2, A1, and Mcl 1 [40, 41]. These antiapoptotic factors work to increase survival of stem cells within the respective tissues to promote growth of cells with protumorigenic potential [42]. Zepp and coworkers, however, found a further alternative pathway through IL-17 receptors A and C dependent Act 1 recruitment of tumour necrosis factor receptor associated factor (TRAF) 4 and subsequent activation of the MAPKs ERK5, ERK1/2, and JNK in colonic stem cells. This provides a core set of cells and the perfect environment for the growth of the tumour.

#### 2.1.2. Tumour Proliferation

IL-17's role in malignancy is not only limited to setting the perfect environment and base for tumour formation but is also responsible for the proliferation of the tumour cells. This has stemmed from evidence showing the involvement of the NF-*κ*B pathway leading to proliferation of synovial cells in rheumatoid arthritis via the mTOR pathway [43]. This is supported by evidence that IL-17 is associated with the increase in proportion of multiple myeloma cells, acting on haematopoietic stem cells resulting in significant expansion of myeloma cells [44]. This may be explained by IL-17's effect on human bone marrow-derived mesenchymal stem cells (hMSCs) where they are able to stimulate the production of reactive oxygen species (ROS) via the activation of Rac1 GTPase and NADPH oxidase 1 (Nox1) and subsequent activation of the MEK-ERK pathway leading to proliferation [45]. Huang et al. also found that the mechanism of activation was very similar to that found in the colonic stem cells used by Zepp et al. involving Act 1 and TRAF 4.

Despite the evidence supporting the proliferative role of IL-17 in cancer, there is counterevidence that shows that IL-17, particularly when added via immunisation, can trigger an antitumour response and cause a reduction in tumour size. This has been demonstrated by immunisation of Th17 cells into a tumour environment trigger activation of cytotoxic CD8^+^ T-cells [46]. The activation of this has been associated with subsequent tumour shrinkage via IL-2 and major histocompatibility complex/peptide (pMHC)-I [47]. This enables the activation of the local immunity through the stimulation of an antitumour T-cell response [48]. Endogenous T-cells have also been shown to be highly effective at reducing tumour size in an* in vitro* model of malignancy where mice are inoculated with malignant cells [49]. However, it is important to note that in both cases, nonautologous Th17 and colon cancer cells were used and therefore may be a less accurate representation of the clinical picture.

#### 2.1.3. Tumour Angiogenesis/Angiostasis

All four characters of inflammation, dolor, calor, rubor, and tumor, could at least in part be accounted for by vascular changes. Karin described how NF-*κ*B and angiogenesis are an important link driving inflammation towards cancer [50] and in the same year Folkman highlighted that the oncogene-driven transition process from inflammation to cancer also influenced the host's prognosis [51]. Angiogenesis has received a lot of attention due to its influence on the tumour grade, metastasis, and therefore patients' prognosis; over-expression of its hallmark cytokine, vascular endothelial growth factor (VEGF), is well known amongst various malignant cancers often with poorer outcomes [52–56]. It is a crucial part of the tumour microenvironment [57] and the literature so seems to map out more pathways of Th17-mediated angiogenesis [25, 27, 33, 51, 57–91] than it does for its inhibition [64, 79, 80, 92–98]. However, the potential to reduce tumour growth via the latter opens doors in the search for potential therapeutic intervention beyond the traditional direct angiogenesis inhibitors for example, bevacizumab (VEGF inhibitor) [99]. This also raises fundamental questions as to why, where, and how such variation takes place. Tumours from different cell lines producing IL-17, the signature cytokine of Th17 cells, conveyed conflicting profiles with regard to angiogenesis. Th17's variable role has also been put down to the different properties of the cytokines it produces. Ultimately, there seems to be missing links in the tumour microenvironment at different tissues that tilts the homeostatic balance of cytokines [100, 101] that push Th17 cells' function towards proangiogenic or proangiostatic outcomes. 

*Proangiogenesis*. High densities of Th17 cells infiltrating tumours have been associated with increased angiogenicity in studies from human subjects, gastric [25], colorectal [87], hepatocellular [82], and pancreatic [86] cancers. The gastric cancer patients whose tumours had higher levels of Th17 infiltration with greater IL-17 and IL-23 mRNA expression suffered from deeper invading disease with higher incidence of lymphatic involvement [25]. Similar to gastric cancer, pancreatic cancer patients' tumours showed a more aggressive behaviour with greater IL-17 and IL-23 expression. Increased expression of VEGF correlating with IL-17 production was found to be an independent factor worsening patients' prognosis [87]. Higher microvascular density (MVD) and lymphocyte infiltration were also seen with IL-17 in hepatocellular carcinoma patients who subsequently exhibited higher mortality rate and reduced survival [82]. Animal models echoed these findings; for example, increased expression of IL-17 in mice transfected with fibrosarcoma cells was associated with tumours exhibiting higher MVD through upregulation of VEGF, keratinocyte-derived chemokine (KC or CXCL1 and CXCL 2), macrophage inflammatory protein-2 (MIP-2), prostaglandins PGE_1_ and PGE_2_, and nitric oxide (NO) production by fibroblasts and stromal cells [66]. The consequence of IL-17's interaction with VEGF production by stromal cells and fibroblasts was associated with enhanced levels of TGF-*β* [74], which in this case was found to promote VEGF-receptor expression on endothelial cells [65]. An important update from Chung et al. [90] revealed Th17 cells forming a paracrine network that induced IL-17-dependent angiogenesis that was also independent of VEGF. Involved in the network were cancer-associated fibroblasts that secrete G-CSF in response to IL-17 via MEK-1/2 signaling and NF-*κ*B; the products recruit MDSC, for example, CD11^+^Gr1^+^ cells [27]. These immature myeloid-derived cells suppress the immune response targeting cancer cell destruction and also promote VEGF-independent angiogenesis [27, 90, 102]. The consequent resistance to conventional chemotherapy is discussed later.

Numasaki et al. also demonstrated IL-17's net angiogenic effects on human nonsmall cell lung cancer (NSCLC) cells in immune-compromised mice via CXCL-8 (IL-8) production and its actions on its receptor (CXCR-2) on endothelial cells via selective production of the following angiogenic cytokines: CXCL-1, CXCL-5, CXCL-6, and CXCL-8 [71]. Among these CXCL-1 is a potent chemoattractant for neutrophils whilst CXCL-5 exert a similar but lesser attraction for neutrophils [79]. It is also known that IL-17 enhances ICAM-1 expression in fibroblasts [24] thus promoting neutrophil adherence and recruitment towards the tumour site. Clinically, neutrophil recruitment has also recently been associated with higher MVD and therefore a more aggressive disease in gastric cancer [91]. IL-17-induced IL-8 release was shown with human cervical cancer [62] and in human glioblastoma cell lines [61]; interestingly the latter study also demonstrated a dose-dependent increase in I*κ*B-*α* mRNA expression, whose product protein is required for NF-*κ*B expression. This transcription factor is well characterized to produce numerous proinflammatory cytokines including IL-6 and IL-8 [103]; an IL-6-mediated promotion of angiogenesis by IL-17 is noted in another* in vivo* study looking at human melanoma [33] and IL-8 is known to be a potent stimulator of angiogenesis and tumour growth [78]. One interesting study also outlined an IL-6-mediated angiogenic cytokine production and tumour growth pathway linked to STAT3 in B16 melanoma and MB49 bladder carcinoma cell lines [33]. Here their findings also outline the independent production of endogenous IL-17 from the tumour cells of IL-17 (−/−) mice. They indicate that IFN-*γ* may have a proangiogenic function since double knockout IL-17 (−/−) and IFN-*γ* (−/−) mice were resistant to tumour growth like their IL-17 (−/−) mice [93]. 

*Antiangiogenesis.* As well as producing the signature cytokine IL-17A (often referred to as IL-17), Th17 cells also secrete IL-17F, IL-21, and IL-22 [101]. Starnes et al. [94] introduced IL-17F's novel role of angiogenesis by depicting a dose-response inhibition of capillary tubule formation* in vitro*; they hypothesised that this may be via TGF-*β* (which in itself is a puzzle by having both pro- and antitumour effects [102]). Subsequently IL-17F has successfully demonstrated direct inhibition of IL-6, IL-8, and VEGF expression of hepatocellular carcinoma (HCC)* in vitro* [97]. It was also associated with reduced vascular cell proliferation. Their immune-deficient murine models' tumours were also smaller with reduced vascularity. A group recently demonstrated reduced CD31^+^ cell infiltration as well as diminished VEGF production with higher levels of IL-17F in an* in vivo *study of colon cancer [98]. Interestingly they could not reproduce the same VEGF patterns* in vitro* reflecting the potential missing pieces of the tumour microenvironment* in vivo*. Therefore continued work is needed to fully map out the mechanism by which tumours switch the IL-17-associated equilibrium in favour of angiogenesis to confer its ability to invade and metastasise, thereby adding further molecular targets for potential pharmacological intervention. Perhaps the context-dependent function of IL-17 [104] calls for work directed towards specific types of cancer.

### 2.2. IL-17 and Tumour Metastasis

Again conflicting reports have arisen from IL-17's role in tumour growth and metastasis. Some IL-17 knockout models showed faster growth and a more aggressive metastatic picture [46, 96]; one of these studies [96] highlighted the potency of endogenous IL-17 in activating the innate immune cells. Here they demonstrated a significant reduction in the number of IFN-*γ*-positive NK, CD8^+^, and CD4^+^ cells within the tumours as well as tumour-draining lymph nodes. Since IFN-*γ* is a potent antitumour cytokine [24, 102], these results suggest that endogenous IL-17 could have a protective role against metastasis. However there are some important mechanisms outlined so far that point to IL-17-mediated promotion of metastasis. Angiogenesis plays an important part in tumour metastasis [24, 57, 101, 102, 105] since basic science dictates that tissues require blood supply for survival at their secondary sites. In addition, the mechanism of lymphangiogenesis is also significant since this promotes spread to lymph vessels [105] by immune cell trafficking [106–108] that includes infiltration by Th17 cells. As clarified by Lohela et al. [109], VEGF-A acting on the receptor VEGF-R2 mediates angiogenesis whilst VEGF-C and VEGF-D are the key cytokines involved in lymphangiogenesis by stimulating VEGF-R3. Intratumoural IL-17 is able to induce the production of VEGF-C and VEGF-D that correlated with increased lymphatic formation as well as worse prognosis in NSCLC patients [110]. Recently gastric cancer also exhibited similar correlation between Th17 cells and cancer metastasis and subsequent prognosis; this study focused on TGF-*β*'s induction on IL-17 expression [91]. Chauhan et al. [111] interestingly displayed in murine cornea (an area normally free from angiogenesis/lymphangiogenesis) how IL-17 was able to induce the expression of VEGF-A and VEGF-C that relied on the presence of IL-1*β* *in vitro.* Their results showed that IL-17 could directly stimulate VEGF-D production on its own whilst IL-1*β* could stimulate VEGF-A and VEGF-C by itself, an effect augmented upon the addition of IL-17. Thus IL-17's positive influence on both angiogenesis and lymphangiogenesis would have contributed for the worst prognosis in patients with the various cancers studied above (in the angiogenesis section).

### 2.3. IL-17 and Chemoresistance

Inhibition of VEGF's proangiogenic actions or antagonising its receptor (mechanism mentioned above) has been the mainstay of adjuvant chemotherapy against a variety of cancers [112]. The effects are however temporary and cancers are known to restart further growth [113]. Suggested mechanisms of forming resistance may perhaps be viewed as similar to antimicrobials. (1) The target cells become resistant to the subject anti-VEGF agent; (2) tumours may begin to proceed with angiogenesis independent of VEGF thereby rendering the agent useless; or (3) invades surrounding tissues and incorporating preexisting blood vessels to feed the tumour [112]. The key study by Chung et al. [90] unveiled a mechanism for anti-VEGF resistance (as introduced above) that confirmed the first two suggestions. Upon treatment with anti-VEGF antibodies the subject tumours recruited higher numbers of CD4^+^ CD8^+^ T-cells (although CD4^+^ numbers were 10 times higher than CD8^+^); specifically the IL-17^+^-IL-22^+^-CD4^+^ (mature Th17) lymphocytic infiltration was associated with higher CD11b^+^Gr1^+^ cells and amplified expression of both G-CSF and IL-17. IL-17 levels were also raised in the peripheral circulation. Taken together the “IL-17-G-CSF” axis was associated with angiogenesis independent of anti-VEGF therapy and promotion of tumour growth with the recruit of immune-suppressive and proangiogenic CD11b^+^Gr1^+^ cells. Contributing to this may be the IL-17-dependent Bv8 secretion produced within tumours despite VEGF-blockade. They also managed to demonstrate how a simple addition of IL-17 to the tumour microenvironment conferred the new resistance, which may suggest that, as far as current knowledge is concerned, IL-17 does not need to rely on other T-cells for developing resistance. It is of note that G-CSFR deficient mice showed similar levels of tumour suppression as those deficient in IL-17RC upon treatment with anti-VEGF antibodies. It is also worth mentioning how IL-17 in this case did not significantly affect the M1 cells and therefore may not be involved in causing VEGF-resistant angiogenesis. A combination treatment involving VEGF blockade as well as invoking IL-17R deficiency reduced tumour growth by almost 80%; however it must be noted that pharmacological inhibition of IL-17A proved to be slightly less effective due to the difficulty in either maintaining or completely eradicating IL-17A signalling.

### 2.4. IL-17 and Tumour Immune-Resistance

#### 2.4.1. Antitumour Effects

Tumour eradications, via a direct cytotoxic effect of Th17 cells, have been shown to exist via the expression of IFN-*γ*; although this effect was similar to Th1 effector cells in relying on IFN-*γ* in immune-resistance against tumours, the effect was significantly greater than that produced by the Th1 cells [114]. A possible indirect mechanism is via the association of IL-17 and the recruitment of tumour-infiltrating IFN-*γ* ^+^ effector T-cells, CD8^+^ T-cells, and NK cells whilst reducing the numbers of T_reg_ cells, thus mediating tumour regression [96].

#### 2.4.2. Protumour Effects

Cancer (besides other pathological diseases) inhibits the normal differentiation of immature myeloid-derived cells into mature cells; thus the population of the immature MDSCs expands [115]. Though copious evidence exploring Th17 cells and their potential tumour suppressing or enhancing effects has been looked at [102, 104, 105], a specific link between the production of IL-17 itself* per se* and effects on tumour immunity has not been clear. He et al. [27] suggested that IL-17 is necessary for the development of MDSCs when comparing IL-17R knockouts and wild type. Functional IL-17 also increased recruitment of MDSCs but reduced CD8^+^ T-cells; these contributed to their finding of enhanced tumour growth with IL-17R (−/−) and IFN-*γ*R (−/−) double-knockout mice that agreed with a previous study [33]. This indicated a potential tumour-promoting role of the IL-17-IFN-*γ* coupling in contrast to other studies in different cancer models [46, 96]. The recently discovered paracrine network of IL-17 also saw an augmented infiltration of MDSCs [90]. The importance of MDSCs' immunosuppressive activity is growing, with higher levels of MDSCs linked with suppression of T-cell activity [115]. Furthermore, as mentioned above, its presence in the tumour microenvironment could be one of the key factors in favouring tumour growth, angiogenesis, and therefore metastasis.

### 2.5. IL-17 as Diagnostic Target

It is well established that early detection of cancer plays a significant part in decreasing morbidity and mortality in patients. With the intricate role IL-17 plays in cancer pathophysiology, evidence is now growing which supports the ability of IL-17 to act as a key diagnostic marker, differentiating between benign and malignant pathology as well as predicting prognosis. It could potentially be a significant tool in allowing early detection.

In lung pathology, differentiating between malignant and benign pleural effusions, caused by a variety of different underlying diseases, is a key factor affecting the management of the patient [116–118]. The diagnosis of a malignant pleural effusion is made via the detection of tumour cells in the pleural fluid. However the likelihood of finding such cells from a pleural fluid cytology (diagnostic method of choice) or needle biopsy is low, with sensitivities ranging between 30 and 60% [119]. In comparison, IL-17 was found to be significantly raised in pleural effusion cytology when detected using enzyme-linked immunosorbent assay (ELISA), [116, 120] with sensitivities as high as 76.8%, increasing to 96.4% when combined with other markers, such as carcinoembryonic antigen (CEA) [116]. In fact, even when testing the serum, patients with malignant serum effusions had significantly higher levels of IL-17 [118, 121]. This provides an alternative diagnostic test for patients with pleural effusions, especially ones of unknown origin.

Similarly, having established a strong connection between multiple myeloma and IL-17, patients suffering from multiple myeloma also display a significant rise in IL-17 levels both in the serum and in bone marrow biopsies [44, 122, 123], with myeloma marrow infiltrating lymphocytes (displaying the IL-17 phenotype) being a strong predictor of lytic bone disease, an effect of the activation of osteoclasts by IL-17 [124].

In oesophageal carcinoma, immunochemical analysis between disease free patients and patients with varying severities of the disease shows a significant strong correlation between IL-17 levels and disease progression in the tissue samples, [125, 126] with Chen et al. showing an associated increase in Th-17 cells in the serum too, again with levels also mirroring disease progression [126]. This could be a potential target for future diagnostic test or to monitor disease progression and response to treatment. However it is worth noting that IL-17 levels as a serum diagnostic marker are not appropriate for all cancer types. Results show that, despite the strong link between IL-17 and inflammatory bowel disease, there is no significant increase in IL-17 levels in patients with colorectal carcinomas [127, 128].

### 2.6. IL-17 as Therapeutic Target

With ever increasing evidence demonstrating the intricate role IL-17 plays in the pathophysiology of cancers, it is inevitable that attention is also now turning towards developing this knowledge into a novel therapeutic method in combating cancer.

Anti-IL-17 monoclonal antibodies are already in existence for the treatment of both inflammatory and immune-mediated conditions; they are listed in Table 1. In the immune-mediated condition psoriasis, using secukinumab (anti IL-17A monoclonal antibody), ixekizumab (anti IL-17 monoclonal antibody), and even an antibody against the receptor itself brodalumab (IL-17RA monoclonal antibody) has been shown to be effective in inducing and maintaining a remittance state in patients with moderate to severe plaques [129–131]. Secukinumab has also been shown to be effective when used to treat the inflammatory condition ankylosing spondylitis [132]. When used in psoriatic arthritis, secukinumab was shown to be both safe and effective in increasing the acute phase reactant and clinical response in patients as well as improving their quality of life [133].

In lung cancer, not only is IL-17 a potential diagnostic marker, but it also may act as a novel therapeutic target. A mice experimental model showed that when anti-IL-17A antibodies were applied locally to the lungs of mice with adenocarcinoma, it induced a reduction in tumour growth, promoted antitumour immunity (increased levels of IFN*γ*, which has been linked to inhibition of proliferation and angiogenesis of tumours), and increased overall survival in the animals [134, 135]. With the emerging roles of IL-17 in lung cancer over time, more experiment is needed to show the potential therapeutic option anti-IL-17 could play in lung cancer management [136]. This inhibition of tumour growth via the administration of an antibody to IL-17 was replicated in a mice model for lymphoma [27] and colonic cancer, [137] with even an abrogated development of metastasis reported as well in a breast cancer mice study [138]. Furthermore, IL-17A knockout mice consistently display a tumour resistance phenotype to a whole variety of cancers, including melanoma, [139] bladder carcinoma, [139], prostate adenocarcinoma, [140] and, with APC genetic predisposition, a decrease in colon tumour initiation [141]. These studies further strengthen the call for more research to ascertain whether anti-IL-17 antibodies could be a potential future cancer therapy, especially as anti-IL-17 monoclonal antibodies are already available and licenced for the use in other diseases.

An alternative way IL-17 could play in cancer treatment is not in the actual targeting of the disease itself, but to assess and monitor the effectiveness of treatment in patients. In ovarian carcinoma, a higher level of IL-17 tumour immune cell infiltration reflects a more chemosensitive tumour to platinum based therapies. At the same time, patients with persistently higher levels of IL-17 tumour immune cell infiltration also indicate the need for a longer course of chemotherapy as these dominated the significant proportion of all recurrences [142]. Similarly, in breast cancer, higher expression of IL-17 was linked with greater probability for recurrence, greater chemotherapy resistance (to docetaxel), shorter disease free survival, and poorer prognosis [143]. In patients with multiple myeloma, serum as well as bone marrow levels of IL-27 decrease whilst IL-17 increases in line with disease progression, with a high IL-27 : IL-17 ratio (i.e., high IL-27 and low IL-17) in the bone marrow associated with better prognosis [144]. These results suggest that IL-17 could potentially be another indicator predicting patient prognosis, thus allowing treatment to be tailored accordingly.

Interestingly, an inverse relationship was found in cervical cancer. A higher risk of recurrence was found in patients who had lower intratumoural levels of IL-17 postradical resection, thus potentially providing a biomarker for patients who may benefit from adjuvant chemotherapy [145]. The monoclonal antibodies in targeting IL-17 at the phase III clinical trial stage are summarised in Table 1.

## 3. Summary

With the prevalence of cancer increasing year to year, it is of utmost importance to find new ways to combat this disease. Here we summarised the ever expanding amount of evidence supporting the intricate role IL-17 plays in the pathophysiology of cancer, from potentially stimulating tumorigenesis, proliferation, angiogenesis, and metastasis, to adapting the tumour in its ability to confer upon itself both immune- and chemotherapy resistance; though often there were opposite views expressed in the literature.

Right from tumourigenesis, IL-17 has been shown to play a role via combination of releasing MDSCs to dampen the body's immune defence system, as well as stimulating proinflammatory cytokines systemically (via the NF-*κ*B pathway) and locally (via CCL2) to maintain an inflammatory environment, resulting in the stimulation of tumour growth via the subsequent expression of antiapoptotic genes and the consequent increased survival of cells with protumourigenic potential. Tumour proliferation was further aided by the IL-17 induced activation of the NF-*κ*B pathway subsequently stimulating the MEK-ERK pathway.

Studies investigating the role of IL-17 in angiogenesis show both a pro- and antiangiogenesis picture. Via the expression of VEGF, IL-17 exerts a proangiogenesis effect, leading to higher microvascular densities and lymphocytes infiltration in certain cancers. A VEGF independent pathway was also found via MEK-1/2 signaling and NF-*κ*B, which leads to suppression of the immune response targeting cancer cells via MDSCs.

Further to its role in angiogenesis, when investigating the effects of IL-17 and tumour metastasis, IL-17 was shown to enhance metastasis via the expression of VEGF inducing both angiogenesis and lymphangiogenesis, subsequently leading to metastasis of tumours. However knockout IL-17 models have also shown a more aggressive metastatic picture, possibly via the loss of the potent antitumour cytokine IFN-*γ* stimulated by IL-17, thus presenting a mixed picture.

However when looking specifically at IL-17F, a subtype of the IL-17 family, an anti-angiogenesis picture was found with decreased levels of VEGF seen. Additionally, there is clear evidence linking IL-17 and the failure of chemotherapy. Via the amplified expression of both G-CSF and IL-17, tumours were able to negate the effects of anti-VEGF chemotherapy. Yet when anti-VEGF therapy was used in conjunction with knocking out IL-17R, a massive reduction in tumour growth was seen.

**Figure 1:**
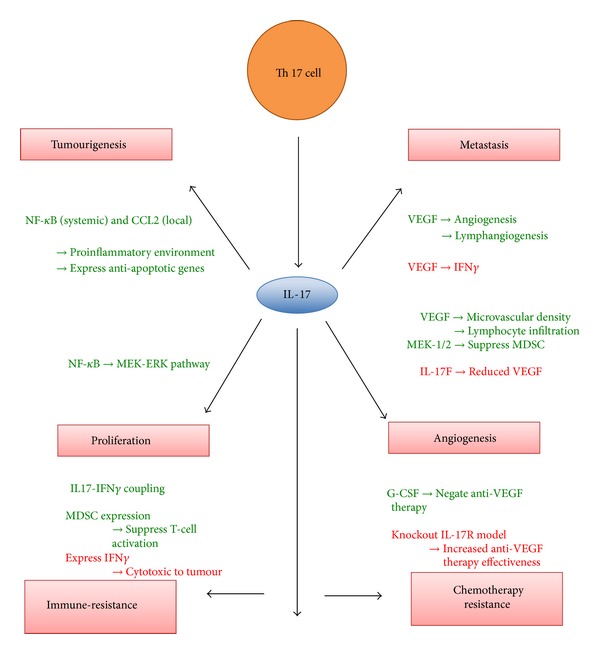
Diagram summarising the reviewed mechanisms by which IL-17 induces (green) or inhibits (red) different aspects of cancer pathophysiology. IL17: interleukin 17; VEGF: vascular endothelial growth factor; NF-*κ*B: nuclear factor kappa-light-chain-enhancer of activated B cells; CCL2: chemokine (C-C motif) ligand 2; IFN*γ*: interferons *γ*; G-CSF: granulocyte colony stimulating factor.

With regard to tumour immune-resistance, again a mixed picture is presented. A protumour environment was demonstrated by IL-17-IFN-*γ* coupling as well as stimulating MDSCs to suppress T-cell activation. Yet an antitumour direct cytotoxic effect was also reported via the expression of IFN-*γ*, thus highlighting an area requiring more research. The above findings have been summarised in Figure 1.

With opinions still often divided on the actual role IL-17 plays in the pathophysiology of cancer, it clearly demonstrates a need for more research in this area. However, even with the current data, IL-17 and its role in cancer pathophysiology do present exciting new options for potential therapeutic and diagnostic targets, which in time will hopefully evolve into novel therapies in the fight against cancer.

## Figures and Tables

**Table 1:** The current clinical trials for monoclonal antibody treatments available in targeting IL-17.

Drug name	Clinical trial	Details
Secukinumab	FIXTURE trial: full year investigative examination of secukinumab versus eTanercept using 2 dosing regimens to determine efficacy in psoriasis.	Phase III trial using 150 mg and 300 mg of secukinumab ClinicalTrials.gov identifier: NCT01358578

Ixekizumab	UNCOVER-2 trial: a multicenter, randomized, double-blind, placebo-controlled study comparing the efficacy and safety of LY2439821 (ixekizumab) to etanercept and placebo in patients With moderate to severe plaque psoriasis.	Phase III trial using 80 mg of ixekizumab ClinicalTrials.gov identifier: NCT01597245

Brodalumab	AMAGINE-2: study to evaluate the efficacy and safety of induction and maintenance regimens of brodalumab compared with placebo and ustekinumab in subjects with moderate to severe plaque psoriasis	Phase III trial using 210 mg and 140 mg of brodalumab ClinicalTrials.gov identifier: NCT01708603
